# The Use of High-Intensity Focused Ultrasound (HIFU) Plus 150mg Bicalutamide as First Line Salvage Therapy for Local Recurrent Prostate Cancer

**DOI:** 10.3389/fonc.2021.705025

**Published:** 2021-11-18

**Authors:** Jian-zhou Cao, Rui Su, Jin-feng Pan, Ze-jun Yan, Qi Ma

**Affiliations:** ^1^ Medical School, Ningbo University, Ningbo, China; ^2^ Comprehensive Urogenital Cancer Center, Ningbo First Hospital, The Affiliated Hospital of Ningbo University, Ningbo, China; ^3^ Department of Urology, Ningbo First Hospital, The Affiliated Hospital of Ningbo University, Ningbo, China; ^4^ Ningbo Clinical Research Center for Urological Disease , Ningbo, China; ^5^ Translational Research Laboratory for Urology, The Key Laboratory of Ningbo City, Ningbo First Hospital, The Affiliated Hospital of Ningbo University, Ningbo, China

**Keywords:** high intensity focused ultrasound (HIFU), anti-androgen therapy, bicalutamide, hypothesis, local recurrent prostate cancer

## Abstract

Patients with localized prostate cancer (PCa) are often treated with radical prostatectomy (RP). However, more than 30% of such patients have high risk of recurrence. Salvage radiotherapy (SRT), androgen deprivation therapy (ADT) and combination of radiotherapy and ADT are the standard care for recurrent PCa. Recently, high intensity focused ultrasound (HIFU) has gradually applied in the treatment of recurrent PCa. Here, we proposed a hypothesis that combined HIFU and bicalutamide 150mg as first line salvage therapy to treat patients with local recurrent PCa with visible lesions due to the following advantages: (1) HIFU is effective in reducing local tumor load, and bicalutamide 150mg is a feasible and safety option to combine with HIFU. (2) Compared with radiotherapy, HIFU plus 150mg bicalutamide is minimal invasiveness with fewer adverse effects and better quality of life(QOL); (3) Radiotherapy can be preserved as the second-line salvage method in the cases who are failure to HIFU and 150mg bicalutamide combination. More clinical trials are warranted to confirm this hypothesis in treatment with recurrent PCa.

## Introduction/Background

Patients with localized prostate cancer (PCa) are often treated with radical prostatectomy (RP), however, more than 30% of such patients have high risk of recurrence after prostatectomy (1–3). Currently, there are several treatment options for patients with recurrent PCa after radical prostatectomy (RP), such as salvage radiotherapy (SRT), androgen deprivation therapy (ADT), and combined radiotherapy with ADT (4–7). A double-blind, placebo-controlled trial (RTOG 9601) has confirmed that the addition of 24 months daily 150mg bicalutamide combined with salvage radiation therapy resulted in significantly higher rates of long-term overall survival (OS) and lower incidences of metastatic PCa and death from PCa than radiation therapy plus placebo in recurrent PCa (8). Therefore, the National Comprehensive Cancer Network (NCCN) published the guideline in 2019 that recommended level 1 evidence supporting 150 mg of bicalutamide daily with radiotherapy combined for two years to treat recurrent PCa (9). However, these therapies also have some adverse effects that may influence the patient’s quality of life (QOL), such as radiation enteritis and cystitis, rectal bleeding, rectal or anal stenosis, and sexual dysfunction. To further avoid these side effects without reducing patient survival benefits, we postulate that high intensity focused ultrasound (HIFU) combined with Bicalutamide 150mg may be another safe and effective treatment option for local recurrent PCa in carefully selected patients with: (1) Visible lesions on imaging; (2) Patients who cannot tolerate the side effects of radiotherapy or refuse to receive radiotherapy.

## Application of HIFU in Treatment of PCa

When HIFU is used for the treatment of PCa, it needs to place a high-power probe in the rectum or urethra to generate acoustic energy focused on the tissue target, which non-specifically ablates tissue through hyperthermia and mechanical effect (10–12). The thermal effects are achieved by heating local tissues to 60°C or higher (usually 1-3mm^2^ area), resulting in near-instantaneous coagulative necrosis and cell death (13). The mechanical effects include cavitation, microstreaming, and radiation forces (11). The mechanical effect would cause cell membrane disruption and then form the cavity, leading the tissue ablation (10).

Under the guideline of the European Association of Urology (EAU), HIFU is described as an investigational treatment modality for PCa treatment (14). Madersbachers has reported 29 cases of PCa who received radical surgery after HIFU treatment, and the pathology showed that HIFU effectively ablated the tumor tissue, which demonstrated that HIFU has a good effect in reducing tumor load (15). Since then, clinicians have tried HIFU for localized PCa and the application of HIFU in early-stage PCa has been successfully written into the guidelines. Whether HIFU plays a role in recurrent PCa after radical prostatectomy and radiation has also been investigated. In the early years, a study reported 4 cases of recurrent PCa after RP were treated with HIFU as first line salvage treatment. These patients were suspected to be local recurrence in the region of the vesicourethral anastomosis with no biopsy, and no bone metastasis or regional lymph node swelling were found before HIFU. At 24-month of follow-up, 2/4 patients were biochemical recurrence-free (defined as an increase in PSA level > 0.2 ng/ml). No major complications were noted (16). Later, Asimakopoulos et al. presented a larger series of salvage HIFU after RP. All included patients were TRUS-evidenced local recurrence with no distant metastasis. During the follow-up, 17/19 patients (89.5%) were classified as success (Success was defined as PSA nadir ≤ 0.1 ng/ml obtained within 3 months from HIFU) (17). In the study of Palermo et al., 22 patients with peri-anastomotic recurrence proved by TRUS were included and received HIFU as first line salvage therapy after radical prostatectomy. None of them showed distant metastasis by bone scan and total body CT scan. 45.5% patients showed a nadir PSA ≤ 0.4 ng/ml three months after HIFU and continue to be considered as a success at a median follow-up of 48 months (18).

In conclusion, though lack of high-quality studies currently, HIFU is a promising method to achieve local tumor control and may be a useful salvage treatment option in recurrent PCa.

## Different Side Effects Between HIFU and Radiotherapy as Salvage Therapy for Recurrent PCa

A small number of studies have shown that HIFU as first-line salvage therapy after RP has obtained satisfactory results and tolerable side effects. In Asimakopoulos’s study, no case of urethrorectal fistula or anastomotic stricture was observed. Two cases of acute urinary retention were resolved with prolonged urethral catheterization. Four cases of stress urinary incontinence were observed; 2 (mild incontinence) were resolved after pelvic floor exercises within 6 months, while 2 cases of severe incontinence required surgical minimally invasive treatmen^17.^ Palermo et al. reported 22% stress urinary incontinence occurred after HIFU treatment. Two sevenths of the patients complained about erectile dysfunction after HIFU treatment. They did not observe cases of urethrorectal fistula or persistent lower urinary tract symptoms (18).

Compared with salvage HIFU, SRT has different complications in patients with recurrent PCa after RP. The side effects of radiotherapy for PCa mainly include gastrointestinal (GI) toxicity, genitourinary (GU) toxicity, Erectile dysfunction (ED) and myelosuppression (19). Lisanne et al. reported long-term treatment toxicity and urinary incontinence rate after radical prostatectomy using SRT. 244 patients were included. Median follow-up after SRT was 50 months. After SRT, *de novo* urinary incontinence complaints (grade ≥ 1) occurred in 6.1% and 17.6% of patients in the acute and late phase. Acute grade ≥ 2 GU and GI toxicity was 19.2% and 17.6%. Late grade ≥ 2 toxicity for GU was 29.9% and for GI was 21.3%, respectively (20). Pirus et al. performed a randomized phase III trial assessing acute toxicity and QOL after SRT. Acute grade 2 and 3 GU toxicity was observed in 13.0% and 0.6% patients with 64 Gy and in 16.6% and 1.7% patients with 70 Gy. Acute grade 2 and 3 GI toxicity was observed in 16.0% and 0.6% patients with 64 Gy, and in 15.4% and 2.3% patients with 70 Gy (21). Myelosuppression occurs less frequently during radiotherapy for PCa and is characterized by leukopenia, neutropenia, anemia, and/or thrombocytopenia (22). ED is also a major complication after radiotherapy, with an incidence of about 28% (23).

Thus, salvage HIFU and salvage radiotherapy have different side effects as local therapy in recurrent PCa. However, it should be noticed that SRT is the mainstream in current studies, and salvage HIFU need more time to accumulate acute and long-term safety data.

## SRT Is Still Effective and Tolerable for Patients Who Are Failure After First Line HIFU Treatment

A study by Filippo et al. intended to evaluate tolerance and acute toxicity in patients who failed to HIFU and salvaged by second line SRT. Acute grade 1 and grade 2 GU toxicities were recorded in 7/15 and 4/15 patients respectively; Acute grade 1 and grade 2 bowel toxicities in 4/15 and 1/15 patients; Acute grade 1 and grade 2 rectal toxicities in 3/15 and 2/15 respectively. No grade 3 or greater acute or late toxicities occurred (24). In another study to treat patients with SRT after HIFU failure, Fernando et al. found that GI toxicity was low. Acute GU toxicity grade ≤ II rate was 45.8%. With only a few patients presenting grade III (8.3%) and grade IV (4.2%) toxicity. Late grade ≥ III GU toxicity was registered in 16.7% of patients. The 3-year disease-free survival rate was 77.8% (25). Julien et al. also evaluated the tolerance and oncologic control with salvage radiotherapy (SRT) after HIFU failure. For the 83 patients treated with exclusive radiation therapy, PFS was 72.5% at 5 year. GI toxicity was low; GU toxicity grade ≤ 2 was 34.5%, grade 3 (4.7%), grade 4 (1.2%), and grade 5 (1.2%). The incidence of severe ED was 14% pre-HIFU, and 51.9% and 82.3% pre-and post-SRT, respectively (26).

These studies suggested that SRT still provides satisfactory oncologic control and with little (or mild) additional toxicity even after HIFU failure.

## The Value of Bicalutamide 150mg in the Treatment of Localized PCa

Bicalutamide is a competitive androgen receptor antagonist that inactivates androgen-regulated prostate cell growth and function, leading to cell apoptosis and inhibition of PCa growth. With an average follow-up of 7.2 years, William et al. found that bicalutamide 150mg plus curative radiation had significantly clinical benefits in terms of OS, PFS and PSA–PFS compared with radiotherapy alone with locally advanced PCa (27). See et al. demonstrated bicalutamide 150 mg significantly reduced the risk of PSA progression, irrespective of whether patients had received radical prostatectomy or radiotherapy as standard care (28). After a median follow-up of 9.7 years, Iversen et al. analyzed the Early Prostate Cancer (EPC) program. This program recruited 8113 patients, among them 4052 patients (49.9%) were randomized to bicalutamide 150 mg and 4061 patients (50.1%) to placebo, in addition to standard care of RT, RP or watchful waiting (W W). At the time of data cut-off, 3032 patients (37.4%) met the criteria for objective disease progression; among them 1483 patients (36.6%) in the bicalutamide and 1549 patients (38.1%) in the placebo group. In the WW group, the median PFS was 6.6 years for patients randomized to receive bicalutamide 150mg compared with 3.7 years for those who randomized to placebo. In addition, bicalutamide reduced the risk of death by 30% in patients with locally advanced disease received RT compared with placebo (29).

## Viability of High Intensity Focused Ultrasound Plus Bicalutamide 150mg Therapy

By virtue of the good effect of HIFU in reducing local tumor load and the good adjuvant effect of 150mg bicalutamide after local treatment, we postulate that HIFU plus bicalutamide 150mg may be a new feasible option for the treatment of recurrent PCa by the following reasons: (1) HIFU is effective in reducing local tumor load, and bicalutamide 150mg is a feasible and safety option to combine with HIFU; (2) HIFU plus 150mg bicalutamide may be a minimal invasive therapy with fewer adverse effects and better QOL; (3) Radiotherapy can be preserved as the second-line salvage method in the cases who are failure to HIFU and bicalutamide combination.

In addition, HIFU has been used as combination therapy to further increase drug delivery efficiency. This is accomplished by increasing blood flow into the heated tumor tissue while also dilating the tumor vessels, further expanding the fenestrae and allowing for greater extravasation (30). Though currently there are no data showed HIFU has synergistic effects with bicalutamide in clinical studies, Bakarev et al. reported that HIFU combined with maximum androgen blocking (flutamide with goserelin) was associated with a significant decrease in numerical density of microvessels in zones of prostate tumor in patients with relapsed PCa (31). In another study, Hahn et al. reported there was no direct correlation between androgen response and heat response, however, it is interesting to see differential response of castration-sensitive and insensitive tumors. Androgen independent PC3 cells were more resistant to high temperatures than androgen dependent VCaP cells. Pre-treatment of VCaP cells with testosterone leads to a more PC3-like kinetic of the heat response (32). As the connection between HIFU and androgen signal pathway is still unclear, more clinical and mechanism related studies could be performed to investigate whether HIFU has synergistic effects with bicalutamide.

## More Considerations

The new generation of hormone therapeutic drugs mainly include abiraterone, an androgen synthesis inhibitor, and AR receptor antagonist such as enzalutamide, apalutamide and darolutamide. Several clinical trials have established these drugs’ value in both metastatic hormone sensitive prostate cancer(mHSPC) and metastatic castration resistant prostate cancer(mCRPC) (33–35). In non-metastatic castration resistant prostate cancer(nmCRPC), AR receptor antagonists also have shown longer OS and PFS in clinical trials (36). In ENZAMET study, ADT combined with enzalutamide was compared with ADT combined with bicalutamide and other first generation of anti-androgen drugs in the treatment of mHSPC directly, and enzalutamide showed significant advantages over bicalutamide and other first generation of anti-androgen receptor drugs (37). However, evidence that supports new hormone therapy agents over bicalutamide in the treatment of local hormone sensitive prostate cancer is lacking (38). Furthermore, currently, most new generation of hormone therapy drugs are combined with ADT during their clinical applications, and only 150mg bicalutamide monotherapy has demonstrated its efficacy in clinical studies (8, 28, 29). More clinical trials should be performed to confirm whether these new hormone therapy drugs with or without ADT are superior over 150mg bicalutamide in local recurrent PCa.

## Conclusion

The hypothesis that HIFU plus 150mg bicalutamide as a safe and effective treatment option for local recurrent PCa in carefully selected patients is promising. The treatment goal is to achieve effective control of local recurrence and avoid distant metastasis, prolong the OS rate and improve QOL. Furthermore, radiotherapy, can be preserved as the second line salvage method in the cases who are failure to HIFU and 150mg bicalutamide combination. We believe that HIFU plus 150mg bicalutamide therapy is theoretically feasible, and further clinical trials are warranted.

## Data Availability Statement

The original contributions presented in the study are included in the article/supplementary material. Further inquiries can be directed to the corresponding authors.

## Author Contributions

All authors listed have made a substantial, direct, and intellectual contribution to the work, and approved it for publication.

## Funding

This study was supported by Zhejiang Natural Science Fund (Grant No.LY20H050002 to QM). Ningbo Social Development Fund (Grant No.202002N3192 to QM), and the Fund of Ningbo Clinical Research Center for Urological Disease (2019A21001).

## Conflict of Interest

The authors declare that the research was conducted in the absence of any commercial or financial relationships that could be construed as a potential conflict of interest.

## Publisher’s Note

All claims expressed in this article are solely those of the authors and do not necessarily represent those of their affiliated organizations, or those of the publisher, the editors and the reviewers. Any product that may be evaluated in this article, or claim that may be made by its manufacturer, is not guaranteed or endorsed by the publisher.
